# Histopathological Changes and Clinical Responses of Buruli Ulcer Plaque Lesions during Chemotherapy: A Role for Surgical Removal of Necrotic Tissue?

**DOI:** 10.1371/journal.pntd.0001334

**Published:** 2011-09-27

**Authors:** Marie-Thérèse Ruf, Ghislain Emmanuel Sopoh, Luc Valère Brun, Ange Dodji Dossou, Yves Thierry Barogui, Roch Christian Johnson, Gerd Pluschke

**Affiliations:** 1 Swiss Tropical and Public Health Institute, Basel, Switzerland; 2 University of Basel, Basel, Switzerland; 3 Centre de Depistage et de Traitement de l'Ulcere de Buruli d'Allada, Allada, Benin; 4 Laboratoire d'Anatomie et Cytologie Pathologiques, Université de Parakou, Parakou, Benin; 5 Centre de Depistage et de Traitement de l'Ulcere de Buruli de Lalo, Lalo, Benin; 6 Fondation Raoul Follereau, Cotonou, Bénin; University of Tennessee, United States of America

## Abstract

**Background:**

Buruli ulcer (BU) caused by *Mycobacterium ulcerans* is a necrotizing skin disease usually starting with a subcutaneous nodule or plaque, which may ulcerate and progress, if untreated, over months and years. During the currently recommended antibiotic treatment with rifampicin/streptomycin plaque lesions tend to ulcerate, often associated with retarded wound healing and prolonged hospital stays.

**Methodology/Principal Findings:**

Included in this study were twelve laboratory reconfirmed, HIV negative BU patients presenting with plaque lesions at the CDTUB in Allada, Benin. Punch biopsies for histopathological and immunohistochemical analysis were taken before start of treatment and after four to five weeks of treatment. Where excision or wound debridement was clinically indicated, the removed tissue was also analyzed. Based on clinical judgment, nine of the twelve patients enrolled in this study received limited surgical excision seven to 39 days after completion of chemotherapy, followed by skin grafting. Lesions of three patients healed without further intervention. Before treatment, plaque lesions were characterized by a destroyed subcutis with extensive necrosis without major signs of infiltration. After completion of antibiotic treatment partial infiltration of the affected tissue was observed, but large necrotic areas remained unchanged.

**Conclusion/Significance:**

Our histopathological analyses show that ulceration of plaque lesions during antibiotic treatment do not represent a failure to respond to antimycobacterial treatment. Based on our results we suggest formal testing in a controlled clinical trial setting whether limited surgical excision of necrotic tissue favours wound healing and can reduce the duration of hospital stays.

## Author Summary

The tropical necrotizing skin disease Buruli ulcer (BU) caused by *Mycobacterium ulcerans* is associated with extensive tissue destruction and local immunosuppression caused by the macrolide exotoxin mycolactone. Chemotherapy with a combination of rifampicin and streptomycin for 8 weeks is the currently recommended treatment for all types of BU lesions, including both ulcerative and non-ulcerative stages (plaques, nodules and edema). Our histopathological analysis of twelve BU plaque lesions revealed extensive destruction of sub-cutaneous tissue. This frequently led to ulceration during antibiotic treatment. This should not be mistaken as a failure of the antimycobacterial chemotherapy, since we found no evidence for the persistence of active infection foci. Large necrotic areas were found to persist even after completion of antibiotic treatment. These may disturb wound healing and the role of wound debridement should therefore be formally tested in a clinical trial setting.

## Introduction

Buruli ulcer (BU), the third most common human mycobacterial disease, is caused by *M. ulcerans* [1], [2]. While the disease is present in around 30 countries worldwide the main focus with the highest prevalence is found in West African countries like Benin, Ghana, Cameroon and the Ivory Coast [3], [4]. Three categories of pre-ulcerative lesions, painless movable subcutaneous nodules or papules, oedema and plaques are distinguished. All three forms of pre-ulcerative lesions may progress to ulcerative lesions, when destruction of the subcutis is leading to the collapse of the overlying epidermis and dermis [1], [5], [6].

In 2004 WHO treatment recommendations for BU changed from a purely surgical treatment to a dual antibiotic therapy with rifampicin and streptomycin for eight weeks [7]. Recurrence rates after antibiotic treatment are low, but a proportion of antibiotic treated patients, in particular those with extensively ulcerated wounds, requires excisions and skin grafting [8]–[13]. During and after completion of antibiotic treatment paradoxical reactions associated with the enhancement of local immune responses and increases in size of lesions may be mistaken as disease progression [14], [15]. Observational studies have shown that while nodules usually heal after antibiotic treatment without further intervention, ulceration and an increase in the size of the lesion is often observed in the case of plaque lesions. These paradoxical reactions may occur after initial improvement and often require extensive medical care, causing long hospital stays. To elucidate the underlying mechanisms and to gain a better insight into the histopathological features of plaque lesions we conducted detailed histopathological and immunohistochemical analyses of tissue specimen from 12 plaque patients treated with rifampicin and streptomycin.

## Materials and Methods

### Ethics statement

Ethical approval (clearance N° 011, 12/10/2010) for analyzing patient specimens was obtained from the provisional national ethical review board of the Ministry of Health Benin, registered under the N° IRB00006860. Written informed consent from the patients or from the guardians of the patients was obtained before surgical specimens were used for reconfirmation of clinical diagnosis and detailed immunohistological analysis.

### Study participants

12 patients aged between five and 70 years (median age 12 years) reporting between April 16 and August 15 2009 with laboratory reconfirmed BU plaque lesions at the Centre de Depistage et de Traitement de l'Ulcere de Buruli d'Allada in Benin were included in the study (Table 1). Most (9/12) lesions were located at the upper (4/12) or lower (5/12) extremities. The diameter of the lesions was between four and 15 cm. All patients were coming from the highly BU endemic Ze commune in the Department Atlantique of Benin. The gender distribution was nine males and three females. Clinical diagnosis was reconfirmed by positive results in at least two of the three laboratory tests (*IS2404* PCR, detection of acid fast bacilli (AFBs) on microscopy and histopathology) performed. All patients completed the WHO recommended combination dual chemotherapy with oral rifampicin (10 mg/kg/day) and i. m. streptomycin (15 mg/kg/day) for 56 days. All patients were tested negative for HIV.

**Table 1:** Characteristics of the 12 Buruli ulcer plaque patients.

Patient	Ulceration during chemotherapy*	Time span between end of chemotherapy and excision	Time span between excision and skin grafting	Time span between skin grafting and discharge	Time span between start of treatment and discharge	Sex	Age (years)	Site of lesion	Lesion size at admission
1	no	no	no	no	61	M	5	thorax	6 cm×4 cm
2	no	no	no	no	55	M	12	trunk	10 cm×7 cm
3	yes	no	no	no	55	M	5	face	7 cm×7 cm
4	no	10	14	16	95	F	13	upper leg	9 cm×8 cm
5	no	10	7	18	90	M	20	foot	5 cm ×4 cm
6	no	10	7	23	95	M	15	elbow	5 cm×5 cm
7	yes	10	7	36	108	F	70	knee	8 cm×5 cm
8	yes	7	7	33	108	M	32	upper leg	15 cm×14 cm
9	yes	17	7	100	179	M	5	lower arm	12 cm×10 cm
10	yes	39	5	31	130	M	12	hand	12 cm×13 cm
11	yes	31	7	36	129	M	12	lower leg	10 cm×8 cm
12	yes	39	7	28	129	F	9	elbow	7 cm×5 cm

*the mean duration from start of treatment to ulceration was 30 days (11–53 days) for those five patients for which beginning of ulceration could be exactly recorded.

### Histopathological analysis

Punch biopsies were taken for histopathological analyses prior to start of chemotherapy on day −2 to day 0 (T1). Of these 12 T1 samples, one was not suitable for immunohistochemical analysis. A second punch biopsy taken 26 to 48 days after start of chemotherapy (T2) became available from 11/12 patients. According to the judgment of the responsible clinician, based on the evolution of the lesions including remaining induration and increasing lesion surface area, nine patients received adjunct surgical treatment seven to 39 days after completion of chemotherapy and had skin grafting five to 14 days after excision. Samples from seven of the nine excised lesions became available for histopathological analysis. In the case of the two other patients that received surgery, a third punch biopsy was taken and analyzed prior to surgical excision. Tissue samples were fixed in 4% neutral-buffered paraformaldehyde for 24 h and subsequently transferred to 70% ethanol for transport. Biopsies were dehydrated, embedded in paraffin, cut into 5 mm thin sections and retrieved on glass slides. After dewaxing and rehydration, sections were stained with haematoxylin/eosin (HE) and Ziehl-Neelsen (ZN) staining of AFBs was performed. Immunohistochemistry was performed with antibodies against Elastase (polymorphonuclear neutrophils [PMNs]; Dako clone NP57), CD3 (T lymphocytes; Dako clone F7.2.38), CD8 (cytotoxic T lymphocytes; Serotec clone 4B11), CD4 (helper T lymphocytes; Dako clone 4B12), CD68 (macrophages/monocytes; Dako clone KP1), Ki67 (proliferation marker; Dako polyclonal rabbit serum) and CD20 (B lymphocytes; Novocastra clone7D1). Staining was performed using Vector NovaRED and haematoxylin as a counterstain.

## Results

### Clinical response to antibiotic treatment

**Figure 1:**
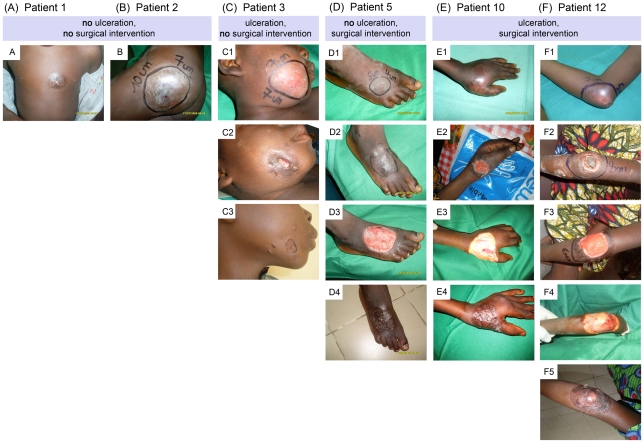
Presentation of BU lesions before and during therapy. Representatives of the different patient groups are depicted. A, B: Patients with lesions that did not ulcerate and which did not require a surgical intervention. C: Patient with a lesion that ulcerated during treatment but did not require a surgical intervention. D: Patient with a lesion that did not ulcerate but was excised; E, F: Patients with lesions that ulcerated during treatment and needed surgical excisions. A: Patient 1; day −1, before start of treatment, punch hole visible. B: Patient 2; day 0, at the start of treatment, punch hole visible. C: Patient 3; C1: day −2 before start of treatment; C2: day 50 after start of treatment; C3: day 300 after end of treatment. D: Patient 5; D1: day −8 before start of treatment; D2: day 10 after end of antibiotic treatment; D3: day 17 after end of antibiotic treatment; D4: day 35 after end of antibiotic treatment. E: Patient 10; E1: day −1 before start of treatment. E2: day 89 after start of treatment. E3: day 39 after end of antibiotic treatment. E4: day 75 after end of antibiotic treatment. F: Patient 12; F1: day 0, at start of treatment; F2: day 26 after start of treatment; F3: day 54 after start of treatment; F4: day 39 after end of antibiotic treatment. F5: day 72 after end of antibiotic treatment.

Included in this study were twelve BU patients (Tab. 1) with single new laboratory-reconfirmed plaque lesions reporting between April 16 and August 15, 2009 at the Centre de Depistage et de Traitement de l'Ulcere de Buruli d'Allada in Benin. All patients received the WHO recommended dual chemotherapy with rifampicin and streptomycin for 56 days [7]. Seven out of 12 lesions ulcerated during chemotherapy (Fig. 1C, E, F). Six of these patients received surgical treatment to remove necrotic tissue (Fig. 1E, F). Based on clinical judgment, tissue was also excised from the lesions of another three patients, which had at this stage not yet developed ulceration, but an induration (Fig 1D). Three patients healed without surgical intervention (Fig. 1A–C), one of them had developed a small ulceration during chemotherapy (Fig. 1C) which needed no further intervention.

For patients who received surgical treatment, excisions were performed 7 to 39 days (average 19 days) after completion of chemotherapy. Skin grafting followed 5 to 14 days (average 8 days) after excision and patients were discharged from hospital 16 to 100 days (average 36 days) after skin grafting. The time interval between start of antibiotic treatment till discharge from hospital was between 55 to 179 days (average 103 days) for the 12 patients (Tab.1). Depending on the location of the lesion this period was prolonged by a phase of physiotherapy (Tab. 1).

### Histopathological features of untreated plaque lesions

**Figure 2:**
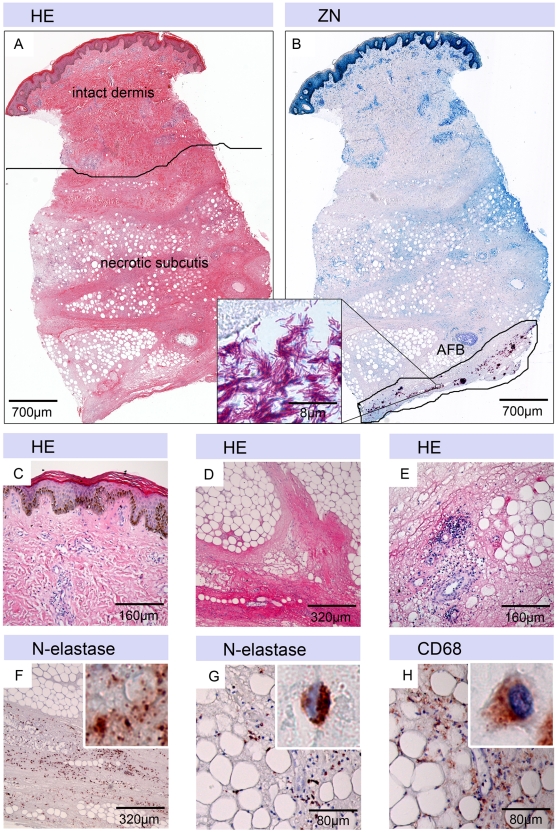
Characteristic histopathological features of tissue samples taken before start of antibiotic treatment. Histological sections were stained either with Haematoxylin-Eosin (HE) (A, C–E), Ziehl-Neelsen (counterstain methylenblue) (ZN) (B) or with antibodies against cell surface or cytoplasmic markers (counterstain haematoxylin) (F–H). A: Punch biopsy with large necrotic areas, fat cell ghosts and oedema but relatively intact epidermis and dermis. B: a band of extracellular AFBs is present in a deep layer of the necrotic subcutis. C: epidermis and dermis. D: necrotic region with fat cell ghosts. E: few infiltrating cells around a blood vessel. F: N-elastase staining revealed the presence of neutrophilic debris inside the necrotic regions. G: few intact neutrophils and H: CD68 positive infiltrating macrophages were found.

For histopathological characterization of the untreated plaque lesions, punch biopsies were taken before start of antibiotic treatment. Certain features, depicted in Fig. 2, were found in all samples analyzed. The dermis presented with relatively intact collagen with minor infiltrations around vessels and glands (Fig. 2A, C), reflecting the pre-ulcerative nature of the plaque lesions. Most strikingly, the subcutis was in all patients extensively necrotic and oedematous (Fig. 2A). Additional features typical for an untreated BU lesion, like fat cell ghosts (Fig. 2D) and minimal infiltration limited to the surrounding of a few remaining partially intact blood vessels (Fig. 2E), were always present. Immunohistochemical staining revealed N-elastase positive neutrophilic debris (Fig. 2F) reflecting an early wave of neutrophilic infiltration. In addition, only few intact neutrophils (Fig. 2G) and CD68 positive macrophages (Fig. 2H) were found. Tissue of only two patients contained also a few intact CD3 positive T-cells in the dermal tissue (data not shown).

Acid fast bacilli (AFB) were found in only 7/31 (23%) of the tissue samples analyzed altogether. This reflects the focal distribution of the mycobacteria and the extension of tissue destruction, attributable to the diffusion of mycolactone, into tissue areas with low mycobacterial burden [16]. Fig. 2B depicts an example, where a mycobacterial focus was sampled. Here a band of extracellular AFBs was found in a deep layer of the necrotic subcutis.

### Persistence of necrotic areas with limited infiltration in the course of antibiotic treatment

**Figure 3:**
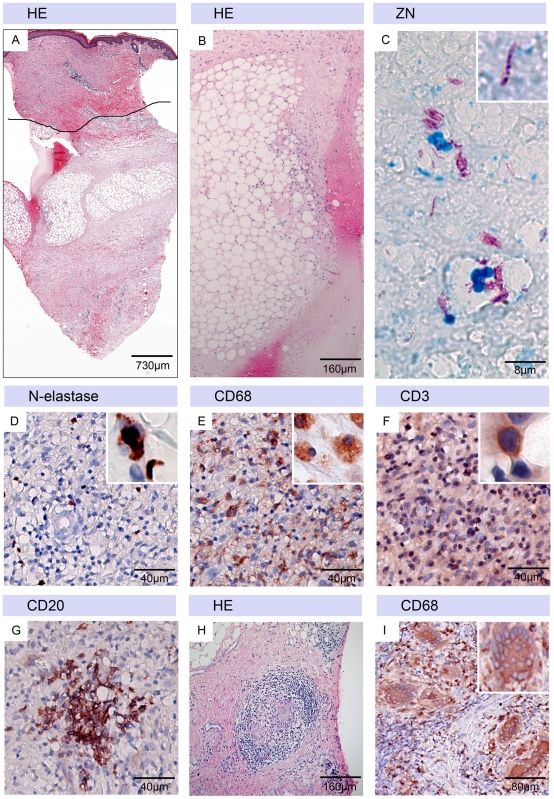
Characteristic histopathological features of tissue samples taken 26–34 days after start of antibiotic treatment. Histological sections were stained either with Haematoxylin-Eosin (HE) (A, B, H), Ziehl-Neelsen (counterstain methylenblue) (ZN) (C) or with antibodies against cell surface or cytoplasmic markers (counterstain haematoxylin) (D–G, I). A: Punch biopsy with large necrotic areas, fat cell ghosts and oedema but relatively intact epidermis and dermis. B: Higher magnification of necrotic tissue with large numbers of fat cell ghosts. C: Small numbers of intra and extracellular beaded AFB. D: N-elastase positive intact neutrophils were rare. E: More intact CD68 positive macrophages and F: CD3 positive T-cells were observed in the dermal tissue. Additionally, small CD20 positive B-cell cluster (G), few granulomas (H) and langhans giant cells (I) were found in only few of the samples.

Punch biopsies taken between 26 and 48 days after start of antibiotic treatment typically consisted still primarily of large oedematous necrotic areas with fat cell ghosts (Fig. 3A/B). Overall, infiltration was much less pronounced than typically found in ulcerative lesions at this time point of antibiotic treatment [17]. 9/11 patients presented with a mild infiltration consisting of very few N-elastase positive neutrophils (Fig. 3D), more CD68 positive macrophages (Fig. 3E) and CD3 positive T-cells (Fig. 3F). These infiltrates were scattered throughout the dermis and extended only in 3/11 samples into the large necrotic areas. Structured infiltrates typically found in healing BU lesions [14], were rare: granuloma formation (Fig. 3H) was found in 2/11 samples, CD20 positive B-cell clusters (Fig. 3G) and giant cells (Fig. 3I) in 3/11 biopsies. AFBs were found in 4/11 samples; they were primarily intracellular or had a ‘beaded’ appearance (Fig. 3C).

### Features of tissue excised after completion of antibiotic treatment

While lesions of three of the enrolled patients healed without adjunct treatment, the responsible clinician decided to support wound healing by surgical excision of affected tissue in 9/12 patients. All nine excisions were performed after completion of antibiotic treatment, 56 to 94 days after start of chemotherapy. While six of the nine tissue samples were excised from lesions, which had spontaneously ulcerated during antibiotic treatment, the other three were excised from non-ulcerated lesions showing no adequate clinical improvement.

**Figure 4:**
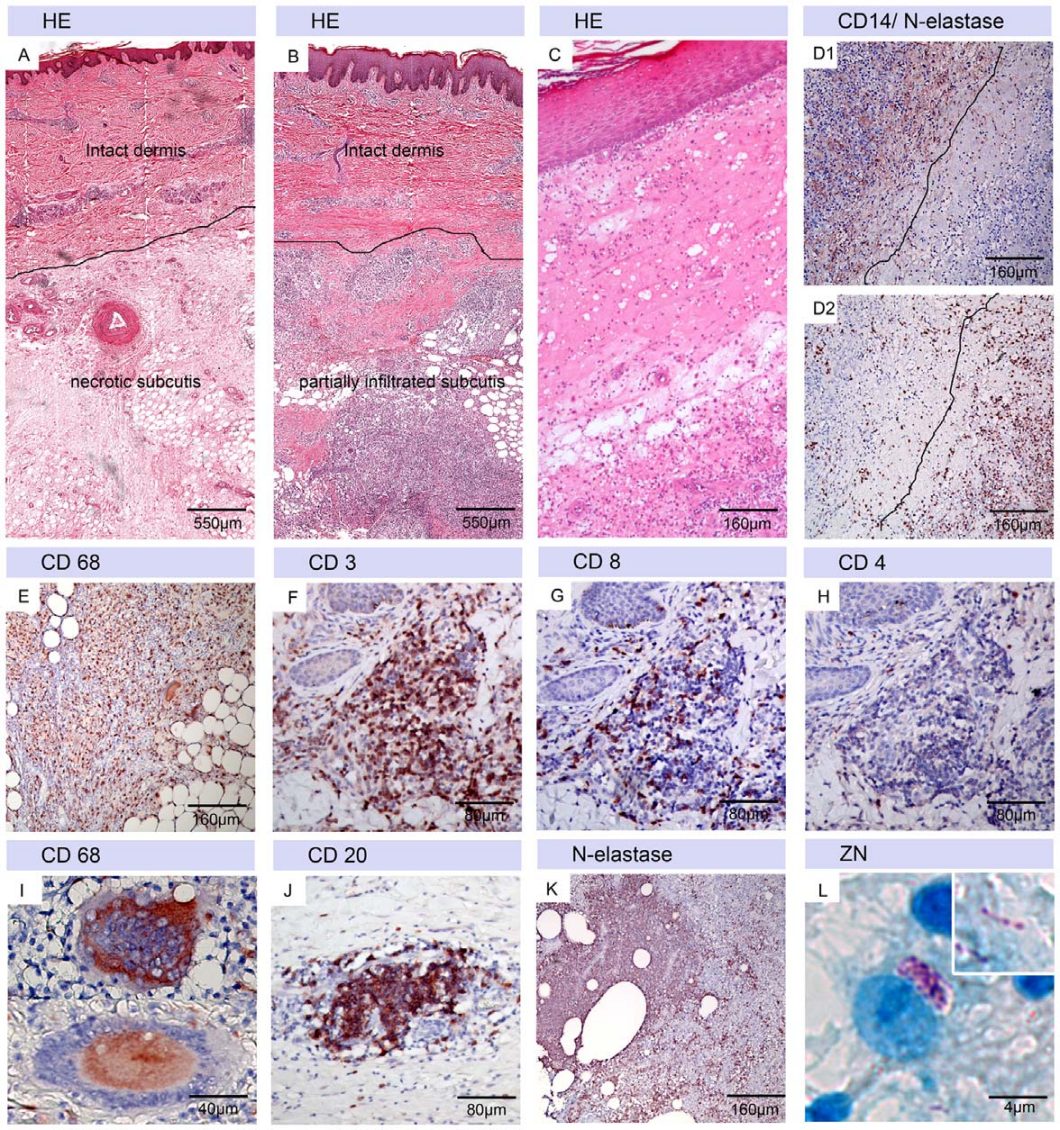
Characteristic histopathological features of tissue surgically excised to support wound healing. Histological sections were stained either with Haematoxylin-Eosin (HE) (A–C), Ziehl-Neelsen (counterstain methylenblue) (ZN) (L) or with antibodies against cell surface or cytoplasmic markers (counterstain haematoxylin) (D–K). A: Overview over an excised tissue specimen still harbouring large necrotic areas with fat cell ghosts and oedema. B: Overview over an excised tissue specimen presenting with mixed infiltration in the former necrotic region. C: Necrosis and oedema of the dermis of an excised non-ulcerative lesion. D: CD14 (D1) and N-elastase (D2) staining revealing a clear border between infiltration with intact CD14 positive macrophages (D1) and neutrophilic debris inside the necrotic area (D2). Infiltrated tissue areas contained large numbers of CD68 positive macrophages (E) and large numbers of CD3 positive cells (F). These belonged mainly of the CD8 (G) and not of the CD4 (H) subset. Langhans and foreign body giant cells (I) and B-cell cluster (J) were present in the majority of the samples. Accumulations of N-elastase positive cells (K) were occasionally found. AFB were rare, had a beaded appearance and intracellular location (L).

All nine excisions consisted to a large extent of necrotic and oedematous tissue with fat cell ghosts (Fig. 4A). In the case of the three patients which had still non-ulcerative lesions at the time of excision, the dermis presented with necrosis and infiltration, indicative for progression towards ulceration (Fig. 4C). Most (7/9) samples showed massive infiltration of the subcutis (Fig. 4B) often with a clear border between intact leucocytes, mainly CD14 positive macrophages/monocytes (Fig. 4D1), and the still necrotic areas containing N-elastase positive neutrophilic debris (Fig. 4D2). Infiltrates were mainly composed of CD68 positive macrophages (Fig. 4E) and CD3 positive T cells (Fig. 4F) with a higher proportion of CD8 positive (Fig. 4G) than CD4 positive (Fig. 4H) T-cells. In addition CD68 positive langhans and foreign body giant cells (Fig. 4I), granulomas and small CD20 positive B-cell clusters (Fig. 4J) were found. Some areas were strongly infiltrated with N-elastase positive neutrophils (Fig. 4K). Angiogenesis inside the necrotic and hypoxic tissue was observed in 5/9 patients (data not shown). Small numbers of intra- and extracellular AFB with a beaded appearance were found in the specimens of 2/9 patients (Fig. 4L).

## Discussion

BU plaque lesions are defined as firm, painless, elevated and well demarcated lesion with more than 2 cm in diameter [18]. Our histopathological analysis of the tissue specimen from plaque lesions reconfirmed earlier quantitative RT-PCR analyses [16], demonstrating a focal distribution of the mycobacteria and a mycolactone-mediated extension of tissue destruction to tissue areas with low mycobacterial burden. Our studies provided no evidence for survival of mycobacterial clusters after chemotherapy. The few AFBs found after chemotherapy had beaded appearance and were largely phagocytosed [17] and all tissues turned culture negative during treatment. Interestingly, in those punch biopsies of untreated BU lesions, where a mycobacterial focus was sampled, mycobacteria were typically found as a band of extracellular AFBs in a deep layer - several mm below the epidermis- of the necrotic subcutis (Fig. 2B). This might explain why in cases, with clinical features consistent with BU, microscopic results are frequently and PCR results are occasionally negative. Especially when collecting diagnostic specimens from non-ulcerative lesions by fine needle aspiration, also deep layers should be sampled to increase chances to reach these bacteria. Focal distribution of *M. ulcerans* and related lack of AFB in some of the tissue samples reflects a major limitation of histopathological analyses using punch biopsies. While findings at a particular location of the lesion may not be representative for the entire lesion, many major histopathological features described here were very consistently found in all samples analyzed.

Also earlier studies [14] demonstrating a reversal of local immune suppression during chemotherapy were confirmed. This process starts with a diffuse chronic infiltration, primarily by macrophages and T cells. While neutrophils play only a minor role in this process, N-elastase positive debris in the necrotic areas are indicative for a wave of neutrophil invasion during the early phase of BU pathogenesis. It has been shown [14] that after an initial phase of diffuse infiltration development of structured leukocyte aggregates, such as B cell clusters and granulomas usually are observed. While the development of such highly organized ectopic lymphoid tissue was also observed in the case of plaque lesions, this was confined to the margins of the necrotic areas. Large regions showing massive coagulative necrosis without significant infiltration were still found in the surgical specimens excised 7–39 days after completion of chemotherapy.

Infiltration and angiogenesis in the affected tissue during and after chemotherapy promotes the resorption of tissue debris. Initial inflammatory responses may be associated with paradoxical reactions, before converging into a phase of wound healing. In two of the 12 patients enrolled, this process of resorption of necrotic tissue was efficient enough to permit healing without ulceration. However, in most cases the necrotic areas of plaque lesions seemed to be too extensive to permit complete resorption without ulceration. In general, our histopathological analysis of plaque lesions revealed a much larger and deeper destruction of the subcutaneous tissue than expected. Spontaneous ulceration during antibiotic treatment was observed in 7/12 patients and tissue samples from three other non-ulcerated patients showed gradual degeneration of the dermis, indicative for a progression to ulceration. While ulceration result in the loss of necrotic tissue, our analysis of tissues surgically excised 7–39 days after completion of chemotherapy from lesions that spontaneously ulcerated during chemotherapy, revealed incomplete loss of necrotic tissue. These findings support the decision of the responsible clinician to support wound healing by debridement of the margins of the ulcers. It is well documented that wound debridement i.e. the removal of materials incompatible with healing, can substantially accelerate the complex wound healing process [19]–[21]. Even if superinfections are controlled with antibiotics, chronic wounds can be caught in a chronic inflammatory phase and debridment is then required to convert the chronic wound bed into an acute wound and mediate healing through the stages of inflammation, proliferation and maturation [20]. This may also apply for BU lesions which show massive infiltration during antibiotic treatment and may subsequently be arrested in a chronic stage without the chance of proper healing. In patients with severe and extensive lesions an early decision for wound debridement may therefore reduce hospital stays to less than 100 days.

In the present study the time span between start of treatment and discharge from the hospital was 55–61 days for the three patients, which did not require an excision. For those three patients, which showed no spontaneous ulceration, but were surgically treated to remove necrotic tissue this time span was 90–95 days. Degeneration of the dermis of the excised lesions harbouring large areas of necrotic tissue indicated that these patients would have developed ulceration at a later stage, leading to a severely delayed subsequent start of the healing process. Those six patients who showed spontaneous ulceration during chemotherapy and received later wound debridement, stayed for 108–179 days in the hospital. Surgical treatment was performed 7–39 days after completion of chemotherapy and skin grafting 5–7 days after excision. Five of these six patients were discharged from hospital 27–38 days after skin grafting, only in one case this time period was much longer (99d) due to secondary infection and delayed wound healing. While bacteria are present on basically every open wound, secondary infections above a critical bacterial load (>10^5^ organisms per gram of tissue) may lead to an arrest of the wound healing process [22]–[24].

Taken together our analysis indicates that due to massive coagulative necrosis only a minority of BU plaque lesions may heal without spontaneous ulceration. It appears advisable to consider debridement of ulcers that have developed in the course of chemotherapy in order to remove necrotic tissue and to favour wound healing. The effect of adjunct surgical treatment should therefore be formally tested in a clinical trial setting to support development of differentiated guidelines for BU wound management.
